# Undifferentiated Intimal Sarcoma of the Inferior Vena Cava with Extension to the Right Atrium and Renal Vasculature

**DOI:** 10.1155/2015/812374

**Published:** 2015-05-28

**Authors:** Aasim M. Afzal, Jamil Alsahhar, Varsha Podduturi, Jeffrey M. Schussler

**Affiliations:** ^1^Department of Internal Medicine, Baylor University Medical Center, 3600 Gaston Avenue, Dallas, TX 75246, USA; ^2^Department of Pathology, Baylor University Medical Center, 3600 Gaston Avenue, Dallas, TX 75246, USA; ^3^Division of Cardiology, Jack and Jane Hamilton Heart and Vascular Hospital, 621 N. Hall Street, Dallas, TX 75246, USA; ^4^Department of Medicine, Texas A&M College of Medicine, Dallas Campus, 3600 Gaston Avenue, Dallas, TX 75246, USA

## Abstract

Primary sarcomas of the great vessels (aorta, pulmonary artery, and inferior vena cava (IVC)) are exceedingly rare. We report a rare case of an undifferentiated intimal sarcoma of the IVC with extension to the right atrium, adrenal, and renal veins. The patient underwent extensive resection, reconstruction of the IVC, and subsequent adjuvant chemotherapy. Patient has tolerated chemotherapy and, at 17 months after resection, the patient remains free of tumor recurrence. Undifferentiated intimal sarcomas remain a rare entity with only five cases of venous undifferentiated intimal sarcomas reported in the literature, two of which occurred in the IVC. Intimal sarcomas tend to carry a poor prognosis with the limited literature available on treatment approaches. Our objective is to highlight this rare entity and possible treatment approach which we utilized. Primary sarcomas of IVC need to be included as part of a complete differential diagnosis in patients with atrial masses or recurrent pulmonary emboli.

## 1. Introduction

Primary sarcomas of the great vessels (aorta, pulmonary artery (PA), and inferior vena cava (IVC)) are seldom seen. IVC sarcomas have a female predominance and are typically present in the fifth decade [1]. These sarcomas are typically leiomyosarcoma or angiosarcoma which carry a better prognosis than undifferentiated sarcoma [1]. We highlight the case of a 52-year-old black female diagnosed with an undifferentiated intimal sarcoma, who then underwent extensive resection with reconstruction of IVC followed by chemotherapy over the past year and a half.

## 2. Case Presentation

A 52-year-old black female with past medical history of hypertension and atrial flutter presented to her primary care physician with a one-month history of shortness of breath, severe dyspnea on exertion, bilateral lower extremity edema, and fatigue. Her blood pressure was elevated at 187/119. Family history included prostate cancer in her father and was not significant for clotting disorders. Home medications included amlodipine, aspirin, digoxin, and metoprolol.

**Figure 1:**
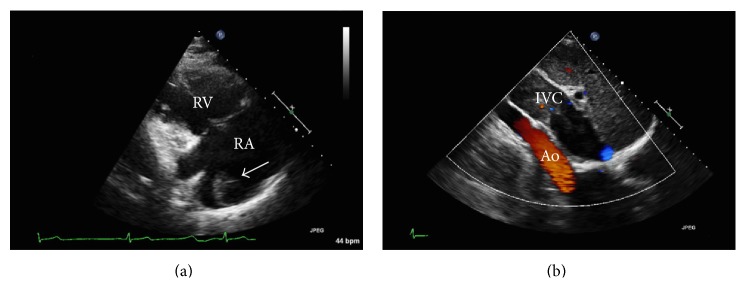
(a) Transthoracic echo showing right atrial mass (arrow). (b) Doppler flow showing IVC tumor restricting blood flow.

A transthoracic echocardiogram (TTE) demonstrated an ejection fraction of 65%, and a 4.5 cm mass was visualized in the right atrium, extending into the IVC and restricting blood flow into the heart (Figures 1(a) and 1(b)). The mass was thought to be either clot or tumor. The patient was started on heparin drip and transferred for higher level of care.

**Figure 2:**
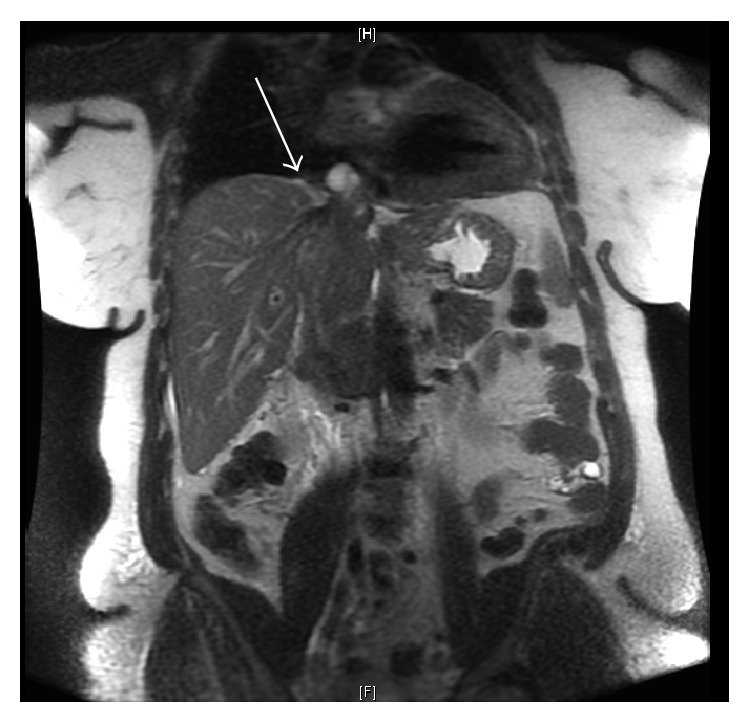
T1 MRI with sagittal view showing the tumor in the IVC with right atrial clot at the tip of the tumor (arrow).

On presentation, she did not appear in distress and was hemodynamically stable. Her lungs were clear to auscultation bilaterally. She had bilateral symmetric 2+ pitting edema of her lower extremities. An abdominal magnetic resonance imaging (MRI) demonstrated a 15.2 × 5.7 × 6.8 cm solid, heterogeneously enhancing tumor of the IVC with extension 4 cm distal to the right renal vein, 1.2 cm into the right renal vein, 1.0 cm into the left renal vein, and upper IVC with a 1.5 cm nodule within the inferior right atrium. The liver was enlarged but no mass extended into the liver or hepatoportal circulation (Figure 2). A cardiac MRI showed tumor within the upper IVC extending to the posterior aspect of the right atrium with a 2 cm thrombus adherent to the intracardiac portion of the tumor (Figure 2). A computed tomography (CT) of chest, abdomen, and pelvis along with an MRI of brain were negative for metastasis.

Due to extensive multiorgan involvement, a multidisciplinary team consisting of cardiothoracic, vascular, and transplant surgeons were involved for a combined cardiothoracic and hepatobiliary surgery. The patient underwent resection of IVC from the right atrium to the infrarenal IVC with reconstruction using a composite graft, right nephrectomy and adrenalectomy, left nephrectomy with autotransplantation in the right iliac fossa, total hepatectomy, and liver autotransplantation. The surgery lasted for about 7 hours with an estimated blood loss of 2 liters. The patient did not undergo sarcoma staging before surgery was performed.

**Figure 3:**
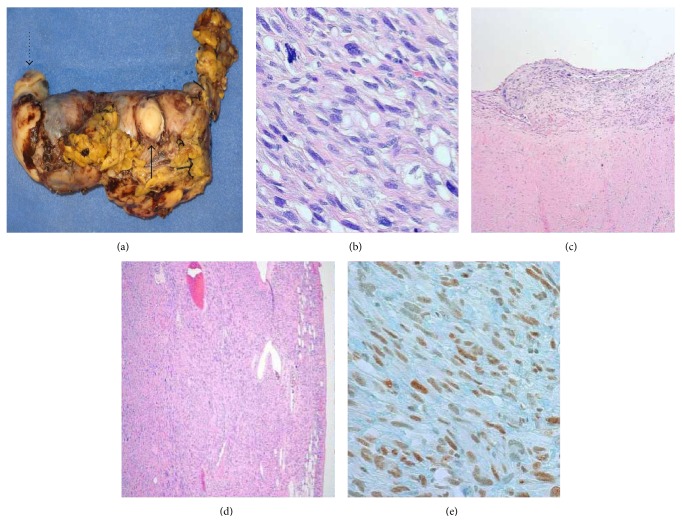
(a) The lumen of inferior vena cava distended by tumor. Dotted arrow specifies cranial end of specimen. Solid arrow indicates ostium of renal vein. (b) Spindled tumor cells in fascicles with nuclear pleomorphism and intracytoplasmic vacuoles (H&E 400x). (c) Neoplastic tumor cells lining the intimal surface of the inferior vena cava (H&E 40x). (d) Tumor invading into adjacent adventitial soft tissue (H&E 40x). (e) Tumor with positive staining for Fli-1 (400x).

Macroscopic examination of the IVC revealed a tan-gray tumor that measured 13.5 × 6.0 × 5.3 cm and completely filled the entire resected specimen. The tumor protruded through the cephalic end of the IVC and the ostium of right renal vein (Figure 3(a)). The cut surface of the tumor was tan-pink with focal areas of hemorrhage and necrosis.

Microscopically, the tumor was comprised of spindled cells in a fascicular growth pattern with cigar-shaped nuclei, intracytoplasmic vacuoles, and exhibited marked nuclear pleomorphism (Figure 3(b)). Mitotic figures were numerous and necrosis was present. The tumor appeared to be arising from the intima of the vessel wall (Figure 3(c)) and focally infiltrated into the adjacent adventitial fibroadipose tissue with no extension to the serosal surface (Figure 3(d)). Immunohistochemistry had negative staining for epithelial differentiation (pancytokeratin AE1-3 and OSCAR), nerve sheath differentiation (S100), endothelial cell markers (CD31, CD34), and smooth muscle markers (smooth muscle actin, muscle specific actin, caldesmon, MYOD-1, and desmin). Tumor cells stained diffusely positive for Fli-1, an endothelial cell marker (Figure 3(e)). Ki-67 or the proliferative index measured 60%.

Given the histologic features and immunohistochemical staining pattern, this tumor was classified as a high grade undifferentiated intimal sarcoma of the IVC. While the margins after resection were free of tumor, the tumor protruded into the proximal and distal ends of the lumen of the IVC as well as into both renal veins.

Postoperatively, patient had an uneventful recovery and was discharged home 7 days after operation. A follow-up echo prior to chemotherapy initiation showed left ventricular hypertrophy with hyperdynamic ejection fraction of 70% but no evidence of residual tumor. She was started on doxorubicin and ifosfamide as the local and distant recurrence rate of a high-grade sarcoma is extremely high. She tolerated the first cycle; however, after the second cycle she developed encephalopathy attributed to ifosfamide. She was switched to a different regimen using gemcitabine and docetaxel, which has been previously utilized in soft tissue tumors. She received multiple cycles of this regimen over the past nine months, developing mild asymptomatic thrombocytopenia and anemia on the new regimen. Surveillance CT over the past year and a half shows that the patient remains free of tumor recurrence. At 19-month status after resection, she remains free of tumor recurrence.

## 3. Discussion

Sarcomas of the IVC have a female predominance and are typically present in the fifth decade [1]. These tumors are most often derived from the medial smooth muscle and are usually leiomyosarcomas, but intimal sarcomas, leiomyomas, synovial sarcoma, angiosarcoma, and rhabdomyosarcoma have been reported [1–4]. Nonmyogenic sarcomas, which are derived from the intima, are even more infrequent and are typically seen in the arterial system, particularly the PA [5, 6]. Intimal sarcomas have also been reported in the superior vena cava (SVC), IVC, and brachiocephalic vein [7, 8]. Typically leiomyosarcoma and angiosarcoma carry a better prognosis than undifferentiated intimal sarcoma with a mean 5-year survival of 33–53% [1].

There have been five cases of venous undifferentiated intimal sarcomas reported in the literature, two of which occurred in the IVC [7, 8]. A clinicopathologic study done by Burke and Virmani reviewed 16 cases of IVC sarcomas. 12 of the 16 patients were women and presented with symptoms of shortness of breath, pain, thrombosis, and IVC syndrome. Of the cases reviewed, 15 of the patients had leiomyosarcoma. Only one case of intimal sarcoma was reported and presented with symptoms of pulmonary embolus. The sarcoma was found to extend the renal and iliac veins. Thrombectomy was performed and the patient did relatively well postoperatively with no complications [1].

Primary sarcomas of IVC need to be included as part of a complete differential diagnosis in patients with atrial masses or recurrent pulmonary emboli. A presentation similar to that of our patient can be seen in patients with leiomyosarcoma, angiosarcoma, renal cell carcinoma with IVC extension, and right atrial thrombus. Clots are seen with tumors of the IVC, as these tumors result in turbulent flow and hemostasis. Only pathology can help differentiate between leiomyosarcoma, angiosarcoma, and intimal sarcoma.

Intimal sarcomas are comprised of epithelioid or haphazardly arranged spindled cells and express endothelial cell markers CD31 and Fli-1. In the case presented, the tumor was made of spindled cells with intracytoplasmic vacuoles and was arising from the intimal wall of the vena cava. The tumor expressed strong Fli-1 positivity, an endothelial marker. Despite not displaying reactivity for other endothelial markers, CD31 and CD34, the tumor was classified as an undifferentiated intimal sarcoma. The pathology results are important as they can affect the prognosis and treatment approach. Histology is important, but the size and grade of sarcoma, as well as adequacy of the resection, determine the need for therapy and outcome. Sebenik et al. suggested that intimal sarcomas are divided into two subtypes, the undifferentiated type and differentiated type [7]. The latter group represents intimal sarcoma of recognized type which may include myxofibrosarcoma, angiosarcoma, epithelioid hemangioendothelioma, and leiomyosarcoma [7]. Undifferentiated types often appear in an older patient population and have a shorter survival than differentiated subtypes [7].

Aggregate data shows poor prognosis for patients with intimal sarcoma, with an average survival of 27 months after chemotherapy and radiation [1]. Another poor prognostic indicator is location within the vessel lumen. Early surgical intervention and complete resection if possible are highly recommended and are the mainstay of therapy [9]. These surgeries are typically extensive in nature and if there is tumor, extension into the heart may require cardiopulmonary bypass with or without hypothermic circulatory arrest [9]. There are no current studies available that give specific guidelines for chemotherapy to treat intimal sarcomas of IVC. We treated our patient based on a meta-analysis done by Pervaiz et al. looking at efficacy of adjuvant chemotherapy for localized resectable soft-tissue sarcomas [10]. That meta-analysis confirmed marginal efficacy of chemotherapy in localized resectable soft-tissue sarcoma with respect to local recurrence, distant recurrence, overall recurrence, and overall survival [10]. The meta-analysis showed that addition of ifosfamide to doxorubicin has a higher antitumor activity on patients with advanced or metastatic soft-tissue sarcoma.

From a detailed literature review, there is record of two other patients who presented with an intimal sarcoma of the IVC and survived past the initial presentation. Our patient tolerated gemcitabine and docetaxel regimen relatively well and follow-up appointments have not documented any recurrences.
